# White Button Mushroom (Agaricus bisporus) Interrupts Tissue AR-TMPRSS2 Expression and Attenuates Pro-inflammatory Cytokines in C57BL/6 Mice: Implication for COVID-19 Dietary Intervention

**DOI:** 10.21203/rs.3.rs-244245/v1

**Published:** 2021-03-25

**Authors:** Shiuan Chen, Xiaoqiang Wang, Desiree Ha, Ryohei Yoshitake

**Affiliations:** Beckman Research Institute, City of Hope; Beckman Research Institute, City of Hope; Beckman Research Institute, City of Hope; Beckman Research Institute, City of Hope

**Keywords:** white button mushroom, androgen receptor, transmembrane protease, serine 2, inflammatory cytokine, myeloid-derived suppressor cells, COVID-19

## Abstract

Transmembrane protease serine 2 (TMPRSS2), an androgen-induced protease associated with prostate cancer, is one putative receptor for coronavirus entry into host cells, where triggering aggressive inflammatory cytokine storm and possibly death in COVID-19 patients. We previously reported that dietary white button mushroom (WBM) antagonized dihydrotestosterone (DHT)-induced androgen receptor (AR) activation and reduced myeloid-derived suppressor cells (MDSCs) in prostate cancer animal models and patients. The present study on C57BL/6 mice revealed that WBM is a unique food that **A)** interrupts DHT induced AR-TMPRSS2 expression in putative COVID-19 targeted organs through its AR antagonistic activity and **B)** attenuates serum pro-inflammatory cytokines which have been implicated in COVID-19 pathogenesis. We hereby propose WBM intake as a potentially low-cost, efficient, and safe dietary intervention to mitigate COVID-19.

## Introduction

Coronavirus disease (COVID-19) is a respiratory and systemic disorder that results from severe acute respiratory syndrome coronavirus 2 (SARS-CoV-2) entry into host cells and its rapid replication. Virus infection triggers immune dysregulation and an inflammatory cytokine storm, ultimately leading to a range of symptoms along the clinical spectrum that include asymptomatic or mild respiratory symptoms, severe lung injury, multi-organ failure, and possibly death [1]. The outbreak of the ongoing COVID-19 pandemic started at the end of 2019 and has continued to spread globally, presenting an unprecedented challenge to the global public health and medical communities [2]. Global efforts have been urgently undertaken to develop prevention and treatment strategies to combat COVID-19, which include vaccine development, drug repurposing, and etc. However, an effective vaccine for immunization or a drug to mitigate COVID-19 lags behind the rapid spread of this virus [3]. This unmet medical need led us to explore the possibility of combating COVID-19 using simple measures such as dietary intervention [4]. The putative use of white button mushrooms (WBM) (*Agaricus bisporus*) in mitigating COVID-19 is based on the findings from our recent studies on prostate cancer models [5] and patients [6].

Accumulating epidemiologic data indicates that the severity and progression of COVID-19 is significantly greater in men than in women [7]. One hypothesis to account for this infection gender discrepancy is that viral entry is potentially enhanced in the lungs and other tissues of men through androgen receptor (AR) regulation [8]. The cellular entry mechanisms of SARS-CoV-2 involve the interaction of a viral spike glycoprotein with transmembrane angiotensin-converting enzyme 2 (ACE2) and transmembrane protease serine 2 (TMPRSS2). The subsequent cleavage of the spike protein by TMPRSS2 is necessary for viral entry [9]. TMPRSS2 was initially characterized as an androgen-regulated gene in the prostate gland and has been associated with prostate cancer [10]. Molecular studies revealed that there are androgen-responsive elements (ARE) within the promoter region of the TMPRSS2 gene, which likely account for its typical androgen-dependent transactivation [11]. The inhibition of the androgen signaling axis impacts TMPRSS2 expression in the prostate gland, as well as throughout the body, including the lungs [12]. Therefore, repurposing anti-AR drugs, in the context of the COVID-19 pandemic, is one of the efforts that are currently being made [13].

Virus entry and replication in host organs trigger a local immune response that recruits macrophages and monocytes to the site of infection, releasing cytokines and priming adaptive T and B cell immune responses [14]. In most cases, this process is well regulated and capable of resolving the infection. However, during a COVID-19 infection, a dysfunctional immune response occurs and triggers an aggressive inflammatory cytokine storm that results in damage to the lungs and multiple other organs, ultimately leading to the possible death of those infected patients [15]. Furthermore, the massive infiltration of mononuclear cells has been detected in infected lungs in addition to the low levels of hyperactive T cells in circulation. The immense migration of innate immune cells to the infected site, initially in order to control viral replication, could contribute the overall tissue damage and eventually lead to multiple organ failures [16]. Therefore, disease severity and clinical outcomes in COVID-19 patients are not only due to the viral load, but also to the host’s response. Novel clinical approaches aimed at balancing the effective immune responses and unsolved inflammation is another major current effort [17].

WBM is a mushroom strain that accounts for 90% of the total edible mushrooms consumed in the United States and many other European and Asia-Pacific countries [18]. A long-term prospective follow-up study in a Japanese cohort suggested that habitual mushroom intake might help to reduce prostate cancer risk [19]. Our clinical phase I trial in prostate cancer patients indicated that oral dietary WBM suppressed prostate-specific antigen (PSA) and reduced the number of myeloid-derived suppressor cells (MDSCs) in blood circulation [6]. We recently showed that chemicals in WBM antagonized dihydrotestosterone (DHT)-induced AR activation and PSA expression in prostate cancer cells and animal models [5]. Other researchers have reported that WBM regulates innate immunity by enhancing NK cell activity [20], promotes maturation of bone marrow-derived dendritic cells [21], and reduces pro-inflammatory cytokine (IL-6, TNFα, interferon-γ) production [22]. β-glucan, the most abundant carbohydrate found in yeast and edible mushrooms, including WBM, is a well-established immune modulator that stimulates the proliferation of lymphocytes while reducing inflammatory factors [23]. Yeast and mushroom-derived β-glucans have also been reported to suppress MDSCs [24] in cancer models, consequently contributing to the enhancement of immunity against tumors [25]. Taken altogether, these findings suggest that chemicals in WBM exert anti-androgen and immunomodulatory effects. We thus hypothesized that dietary intake of WBM may act as a unique nutritional intervention for COVID-19 by suppressing TMPRSS2 expression, via the AR signaling pathway, and reducing pro-inflammatory factors.

Hereby, we conducted experiments using either dihydrotestosterone (DHT) pellet-bearing or untreated C57BL/6 male mice to test our hypothesis. We report the results from our proof-of-concept study that dietary WBM interrupt AR-induced TMPRSS2 expression throughout the body, inclusive of the putative COVID-19 target organs (lungs, small intestine and kidneys). We also report that oral intake of WBM decreases pro-inflammatory factors and reduces the MDSC counts in both blood and spleen.

## Results

### WBM Suppresses DHT-induced AR-TMPRSS2 Expression in Putative COVID-19 Impacted Organs in Mice

We recently reported that dietary WBM antagonized DHT-induced AR activation and PSA expression in prostate cancer models and in mouse prostate glands, without observable toxicity [5, 6]. We thus hypothesized that dietary WBM may interrupt DHT-induced AR-mediated TMPRSS2 expression throughout the body, including the putative COVID-19 targeted organs (lungs, small intestine, and kidneys). C57BL/6 mice were used to test our hypothesis.

**Figure 1:**
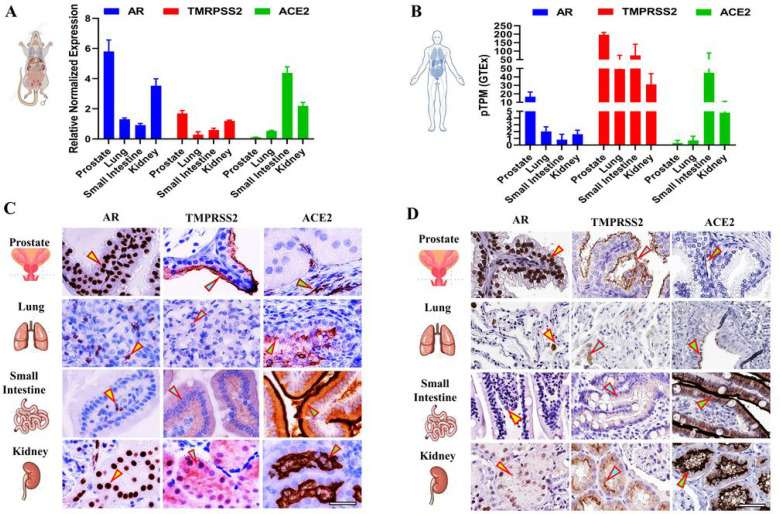
Tissue expression and distribution of AR, TMPRSS2, and ACE2 in mice versus in humans. A) The relative mRNA expressions of Ar, Tmprss,2 and Ace2 in prostate, lungs, small intestine, and kidneys in 8-week-old intact male C57BL/6 mice were quantified by qRT-PCR. B) The mRNA expression levels of Ar, Tmprss2, and Ace2 in prostate, lungs, small intestine, and kidneys in adult humans. TPM: transcripts per million; pTPM: all TPM values per sample scaled to a sum of 1 million TPM. C) Immunohistochemistry (IHC) of AR, TMPRSS2, and ACE2 in prostate, lungs, small intestine, and kidneys in 8-week-old intact male C57BL/6 mice; D) IHC of AR, TMPRSS2, and ACE2 in prostate, lungs, small intestine, and kidneys in adult humans. Scale bar of representative images is 40X by 100 μm.

We first determined the basal mRNA levels of *Ar, Tmprss2,* and *Ace2* in eight-weeks-old male C57BL/6 mouse tissues verus in adult human tissues. Their basal levels were different in mouse tissues: *Ar* levels were highest in the prostate followed by the kidneys, *Tmprss2* levels were highest in the kidneys, and *Ace2* levels were highest in the small intestine (Fig. 1A). A similar expression pattern was also observed for *Ar* and *Ace2* in human tissues, but for *Tmprss2,* the highest levels were found in the prostate (Fig. 1B). We next performed IHC to identify the specific cell types present in the tissues that displayed expressions for AR, TMPRSS2, and ACE2 in mice (Fig. 1C) verus in humans (Fig. 1D). Similar to mRNA expression, AR, TMPRSS2, and ACE2 proteins were detected in all of the tested tissues at varying degrees. Mouse prostate glandular epithelial cells showed a high level of staining for AR (nucleus) and TMPRSS2 (apical lumen surface). A small proportion of basal cells that lie beneath the prostate epithelium exhibited a low level of ACE2 staining. The smilar expression patterns of AR, TMPRSS2, and ACE2 were also observed in human prostate (Fig. 1C/D - Prostate, indicated by arrows). In mouse lungs, a strong staining for ACE2 was detected exclusively in the lung respiratory bronchiole epithelium, while moderate staining for AR and weak staining for TMPRSS2 was detected in alveolar cells, which is consistent with the expression pattern observed in human lungs (Fig. 1 C/D - Lung, indicated by arrows). The mouse small intestine mucosa showed an intense staining of ACE2 on the surface of the enterocytes, located on the top part of the villi. TMPRSS2 displayed a moderate staining in the crypt and the lower portion of the villi, with the exception in the goblet cells, with the staining gradually diminishing towards the top of villi. Meanwhile, AR staining was observed predominantly in lamina propria. These expression patterns mirror the results from human small intestinal tissue (Fig. 1C/D - Small Intestine, indicated by arrows). Concerning mouse and human kidneys, the AR staining was observed in the nuclei of tubular cells. TMPRSS2 was mainly expressed in the proximal tubular cells, while ACE2 was observed on the apical surface of the tubular cells (Fig. 1 C/D - Kidney, indicated by arrows).

**Figure 2:**
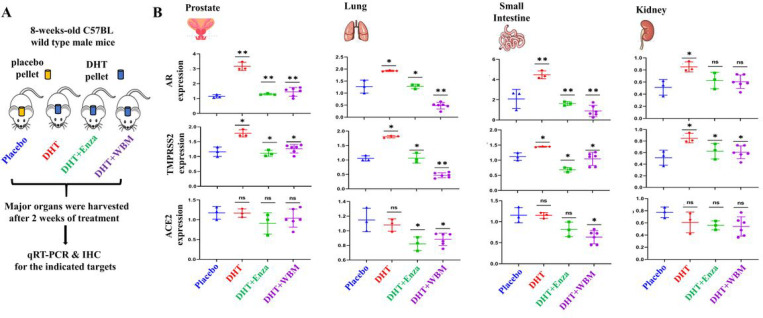
WBM suppresses DHT-induced AR-TMPRSS2 expression in putative COVID-19 impacted organs in mice. A) 8-weeks-old male C57BL/6 mice were subcutaneously grafted pellets (3 mice with placebo pellets versus 12 mice with DHT pellets). The 3 mice with placebo pellets were gavaged daily with 100 μL PBS for 2 weeks (Placebo group). The 12 mice with DHT pellets were randomly divided into 3 groups and treated daily for 2 weeks as follows: 3 mice with 100 μL PBS (DHT group), 3 mice with 300 μg enzalutamide (DHT+Enza group), and 6 mice with 6 mg WBM (DHT+WBM group). B) The mRNA levels of Ar, Tmprss2, and Ace2 in prostate, lungs, small intestine, and kidneys of each group were quantified by qRT-PCR. p values were determined by multiple Student’s t-tests. (*p<0.05, **p<0.01, ***p<0.001. ns. No significant difference.)

Based on the above observations, we designed animal experiments (Fig. 2A) to validate that in putative COVID-19 targeted organs, the expression of TMPRSS2 was up-regulated in the presence of DHT and to determine whether WBM intake antagonized DHT-induced TMPRSS2 expression in these organs. Eight-weeks-olds male mice bearing placebo pellets were used as a baseline control to compare to the mice bearing DHT pellets. Enzalutamide, a well-defined AR antagonist, or WBM were given to DHT-treated mice as treatments. After two weeks of treatment, we harvested lungs, kidneys, small intestine, and prostate from each mouse for qRT-PCR and IHC analysis. Prostate was used as a positive control for systemic AR agonistic and antagonistic responses. As expected, *Ar* and *Tmprss2* expression was androgen-responsive in the prostate, as shown in DHT pellet-bearing mice, which were suppressed by treatment with either enzalutamide or WBM (Fig. 2B - Prostate). Except for the kidneys, which showed a minimal effect on DHT treatment, DHT induced the expression of *Ar* and *Tmprss2* in both the lungs and the small intestine, while enzalutamide or WBM antagonized DHT-induced expression in the same organs. The expression of *Ace2* did not display androgen responsiveness in the prostate and the kidneys. However, *Ace2* expression displayed a trend of downregulation in the lungs and small intestine by enzalutamide or WBM (Fig. 2B - Lung, Small Intestine and Kidney).

**Figure 3:**
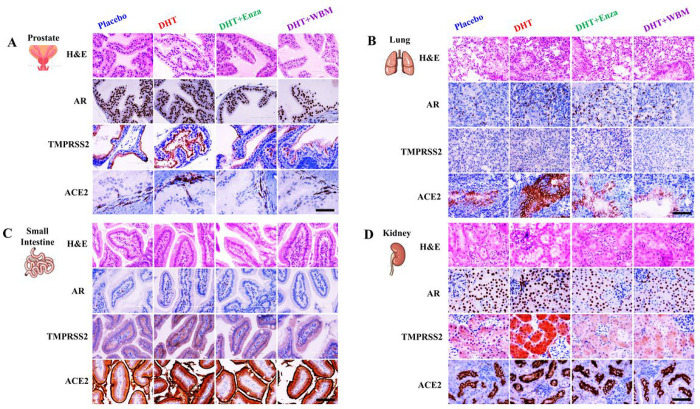
Histological evaluation of AR-ACE2-TMPRSS2 expression in putative COVID-19 impacted organs in response to DHT or WBM treatments. 8-weeks-old male C57BL/6 mice were subcutaneously grafted pellets (3 mice with placebo pellets versus 12 mice with DHT pellets). The 3 mice with placebo pellets were gavaged daily with 100 μL PBS (Placebo group). The 12 mice with DHT pellets were randomly divided into 3 groups and treated daily for 2 weeks as follows: 3 mice with 100 μL PBS (DHT group), 3 mice with 300 μg enzalutamide (DHT+Enza group), and 6 mice with 6 mg WBM (DHT+WBM group). Hematoxylin and eosin (H&E) and IHC of AR, TMPRSS2, and ACE2 in A) prostate, B) lungs, C) small intestine, and D) kidneys in each group. Scale bar of representative images is 20X by 200 μm.

We also performed IHC to identify the specific cell types that displayed altered expressions for AR, TMPRSS2, and ACE2 upon AR agonist or antagonist treatments in mice (Fig. 3). The hormone-responsive prostate glandular epithelial cells showed an increased number of AR positive glandular epithelial cells (nucleus) and TMPRSS2 (apical lumen surface) proteins in response to DHT when comparing the Placebo and DHT groups. This increase was suppressed by enzalutamide or WBM, as seen when comparing the DHT group to DHT + Enza/DHT + WBM groups. ACE2, present in basal cells that lie beneath the prostate epithelium, displayed moderate changes from each treatment (Fig. 3A - Prostate). In mouse lungs, the staining intensity of AR and TMPRSS2 in alveolar cells, especially ACE2 in lung respiratory bronchiole epithelium, was increased upon DHT treatment, which was reduced by enzalutamide or WBM treatment (Fig. 3B - Lung). DHT induced the expression of AR, TMPRSS2, and ACE2 in the small intestine mucosa, but the addition of enzalutamide or WBM was able to suppress these DHT-induced expressions (Fig. 3C - Small Intestine). Concerning the mouse kidneys, DHT treatment increased the intensity of the AR staining, while enzalutamide or WBM displayed a slight trend in attenuating DHT-induced AR expression. On the other hand, TMPRSS2 was induced significantly upon DHT exposure and was suppressed by enzalutamide or WBM. We did not observe visible changes upon AR agonist or antagonist treatments (Fig. 3D - Kidney).

Taken altogether, our analyses clearly demonstrate that AR agonist or antagonist treatments have an effect on the expression of AR and TMPRSS2, particularly in the lungs, small intestine, and kidneys. Slight changes to ACE2 by AR agonist or antagonist on lungs and small intestine were also observed. Importantly, we experimentally demonstrated that WBM intake in mice disrupts DHT-induced AR-TMPRSS2 expression throughout the body, including the putative COVID-19 target organs (lungs, small intestine, and kidneys). Our findings pave a path for WBM to be utilized as a new dietary-based prevention or therapeutic option.

### WBM Attenuates Pro-inflammatory Cytokines and Reduced MDSCs in Mice

**Figure 4:**
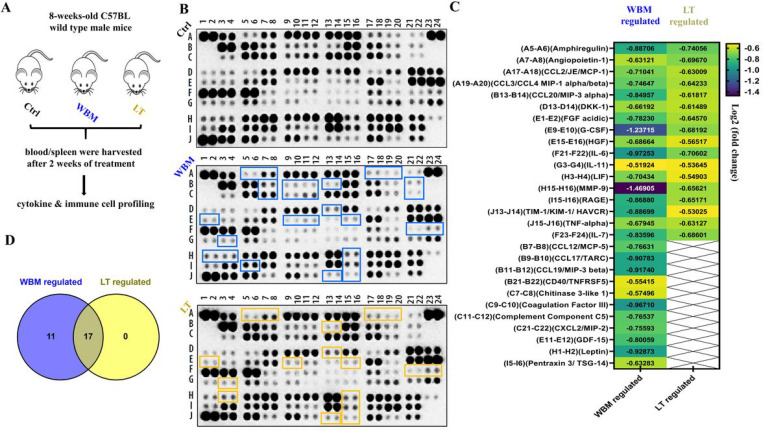
WBM attenuates pro-inflammatory cytokines in the serum of mice. A) 15 intact C57BL/6 mice were randomly divided into 3 groups and treated daily for 2 weeks as follows: 5 mice in Ctrl group were gavaged with 100 μL PBS, 5 mice in Letinan (LT) group were gavaged with 6 mg Lentinan, and 5 mice in WBM group were gavaged with 6 mg WBM. B) Proteome Profiler Mouse XL Cytokine Array Kit was used to semi-quantify 111 mouse cytokines, chemokines growth factors, and other soluble proteins in the serum from each group. The image of dot spots represents the changing levels of cytokines. WBM-regulated cytokines were labeled with a purple-blue color, while LT-regulated cytokines were labeled with a light yellow color. C) The Quick Spots software was applied to measure the mean spot pixel density and significant changes (treatments versus control) were defined as up-regulated (log2 fold change ≥ 0.5, p<0.05) versus down-regulated (log2 fold change ≤ 0.5, p<0.05). D) The overlapping cytokines between WBM versus LT treatment were displayed by Venn diagram using Venny 2.1.

β-glucans are glucose polymers with a backbone of linear β−1,3-linked D-glucose molecules (β−1,3-D-glucan). They exist most commonly as cellulose in plants, the cell walls of yeast, and as certain fungi and mushrooms [26]. β-glucans derived from either yeast or mushrooms were reported to enhance immunity against tumors or viruses [27]. Our clinical phase I trial in prostate cancer patients suggested that oral dietary WBM decreased a portion of MDSCs in peripheral circulation [6]. In line with these observations, we hypothesized that dietary WBM could suppress inflammatory factors and reduce MDSCs via β-glucan. In the present study, we designed an *in vivo* study (Fig. 4A) to investigate the immunoregulatory effects of WBM. Eight-weeks-old male mice were treated with WBM or LT, a shiitake mushroom derived β-(1, 3)-glucan, while untreated mice were used as a control. After two weeks of treatment, we collected serum for cytokine profiling and isolated blood and spleens for MDSC characterization.

By performing the Mouse XL Cytokine Array, which contains 111 mouse cytokines, chemokine growth factors, and other soluble proteins, we identified that a set of factors were down-regulated (log2 fold change ≤ 0.5, p < 0.05) by WBM or LT, as compared to the Ctrl, while up-regulated factors (log2 fold change ≥ 0.5, p < 0.05) were not observed after both treatments (Fig. 4B/C). WBM suppressed the levels of 28 cytokines and LT suppressed 17 cytokines. All 17 cytokines down-regulated by LT were also down-regulated by WBM (Fig. 4D). LT is the shiitake mushroom-derived immunomodulatory β-(1, 3)-glucan; the same form of β-glucan was also found as the most abundant carbohydrate in WBM [28]. Our results indicate that β-glucan is probably a major component of WBM that modulates immune function. The common 17 cytokines suppressed by both WBM and LT (Fig. 4C/D) include molecules that have been implicated in COVID-19 pathogenesis such as IL-6, IL-7, CCL-2/MCP-1, CCL-3/CCL-4 MIP-1α/β, TNF-α, and G-CSF. The other 11 cytokines (Fig. 4C/D) were regulated by just WBM, not LT. Among these, we found a defined cytokine trait (CCL-12/MCP-5, CCL-19/MIP-3β, CXCL-2/MIP-2, et al) of severe COVID-19 outcome [29, 30]. To further examine the identified cytokine profiles and the pathways underlying their synergism, we assessed the pathways by unbiased Gene Set Enrichment Analysis (GSEA) (Table 1). 28 WBM-regulated cytokines (including 17 common regulated factors between WBM and LT) were analyzed by the GSEA software and the WIKI pathways gene sets were selected. More importantly, Lung_Fibrosis and COVID19_Adverse_

Outcome_Pathway were identified as the top two regulated pathways among Top5 pathways (Table 1). An cytokine trait that includes 11 factors (IL-6, TNF-α, G-CSF, MMP-9, HGF, FGF acidic, CCL-2/MCP-1, CCL-3/CCL-4 MIP-1α/β, CXCL-2/MIP-2, and Pentraxin 3) was associated with the lung fibrosis pathway, while a subset of 6 cytokines (IL-6, TNF-α, G-CSF, CCL-2/MCP-1, CCL-3/CCL-4 MIP-1α/β, IL-7) was related to the COVID-19 adverse outcome pathway.

**Figure 5:**
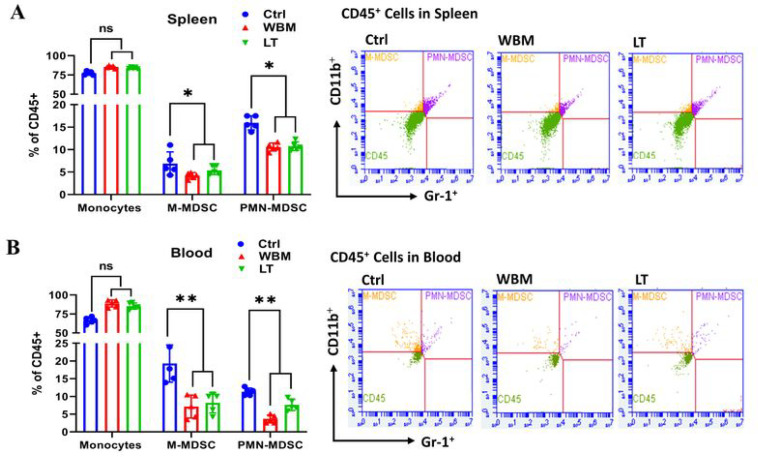
WBM decreases MSDC counts in spleen and blood of mice. BD Accuri C6 Plus flow cytometer was used to identify myeloid-derived suppressor cells (MDSCs) in A) spleen and in B) blood. BD Accuri C6 system software was used to analyze the data. Mouse monocytic MDSCs (M-MDSCs/CD45+/CD11b+/Gr-1low/mid) and granulocytic MDSCs (PMN-MDSCs/CD45+/CD11b+/Gr-1high) were gated to show the two population of MDSCs. p values were determined by multiple Student’s t-tests. (*p<0.05, **p<0.01, ***p<0.001. ns. No significant difference.)

To further characterize the immune responses in relation to WBM treatment, we quantified the levels of MDSCs in spleen (Fig. 5A) and blood (Fig. 5B) by performing multi-parametric FACS analysis. WBM or LT clearly decreased the total counts of M-MDSCs (CD45^+^/CD11b^+^/Gr-1^low/mid^) and PMN-MDSCs (CD45^+^/CD11b+/Gr-1^high^) in both peripheral blood and spleen (Fig. 5). MDSCs have been described to be highly increased in COVID-19 patients [31]. An emerging study revealed that PMN-MDSCs expand during COVID-19 infection and are correlated with IL-1β, IL-6, IL-8, and TNF-α plasma levels, particularly in patients who required intensive care treatments, suggesting new therapeutic options geared towards MDSCs [32]. Taken altogether, our analyses demonstrated that WBM has immunoregulatory functions on cytokine profiles and MDSC regulation. Particularly, we observed a significant decline of cytokine profiles associated to lung fibrosis and COVID-19 adverse outcomes.

## Discussion

TMPRSS2 and ACE2 are two putative receptor proteins for the virus, SARS-CoV-2, to gain entry into host cells [9]. TMPRSS2 was first characterized as an androgen-regulated gene in the prostate gland [10, 11], but emerging studies have recently demonstrated that TMPRSS2 is also positively regulated by androgen in murine lungs and in human lung cells [12]. Since TMPRSS2 is implicated in COVID-19 pathogenesis, it supports a promoting role of androgens that males, relative to females, are disproportionately affected by COVID-19 in terms of mortality and morbidity [8]. Therefore, repurposing anti-androgenic drugs in the context of the COVID-19 pandemic is one of the major efforts currently being made [13]. Several preclinical studies, including some employing large epidemiological cohorts, suggested that blocking androgen signaling might protect against COVID-19 [33, 34]. The clinical trial using AR antagonists, proxalutamide/GT0918, in COVID-19 is currently underway (ClinicalTrials.gov, NCT04446429. Anti-Androgen Treatment for COVID-19). The trial released the preliminary analysis of proxalutamide as a treatment for COVID-19 patients. The data showed that proxalutamide could significantly ameliorate symptoms and prevent hospitalization for COVID-19 patients [35]. Putative use of WBM in mitigating COVID-19 was supported by findings from our clinical phase 1 trial in prostate cancer patients, as well as from the very recent preclinical studies that address the anti-androgen receptor activity of WBM. Our clinical phase I trial in prostate cancer patients indicated that oral dietary WBM suppressed circulating PSA levels [6]. We recently showed that dietary WBM antagonized DHT-induced AR activation and PSA expression in prostate cancer models and mouse prostate glands [5]. Considering these findings from our clinical and preclinical studies, dietary WBM could antagonize DHT-induced AR activation and TMPRSS2 expression throughout the body, including the putative COVID-19 targeted organs of lungs, small intestine, and kidneys.

In the current study, we demonstrated that androgen regulates the expression of AR and TMPRSS2 in subsets of pulmonary and intestinal epithelial cells. The AR and TMPRSS2 levels are markedly elevated in the lungs and the intestine upon DHT exposure, while WBM and an AR antagonist, enzalutamide, effectively repressed DHT-induced transcriptions of AR and TMPRSS2 (Figs. 2 and 3). To date, there are debates regarding the androgen-induced TMPRSS2 regulation in lung epithelial cells. A preprint article suggested no evidence for increased TMPRSS2 expression in the lungs of males, compared to females, in humans and mice. In their male mouse model, treatment with enzalutamide did not decrease pulmonary TMPRSS2 [36]. However, several other studies demonstrated with strong evidence that androgen regulates the expression of TMPRSS2 and AR in subsets of lung epithelial cells, and AR antagonists inhibit SARS-CoV-2 infections in vitro [37, 38]. Another preprint article also demonstrated the co-expression of AR and TMPRSS2 in specific lung cell types. Treatment with enzalutamide reduced TMPRSS2 levels in human lung cells [39]. As compared to the numerous investigations of AR-TMRPSS2 in lungs, very few research studies document this regulation in the other putative COVID-19 target organs, such as the small intestine and kidneys [40]. There was one preprint article that noted the reduced TMPRSS2 staining in the bronchial epithelium of the lungs, columnar epithelium of small intestine, and proximal convoluted tubules in the kidneys of androgen-deprived C57BL/6 mice from castration [41]. In our experiments, we observed that AR and TMPRSS2 levels were significantly elevated in the lungs and the small intestine upon DHT exposure, while intake of WBM and enzalutamide effectively repressed DHT-induced transcriptions of AR and TMPRSS2 in multiple organs. Our study using a male mouse model provided additional evidence that AR induced TMPRSS2 expression in the putative COVID-19 targeted organs of lungs, small intestine, and kidneys. By the same principle as the ongoing clinical trial with AR antagonists for COVID-19, our studies suggest WBM to be a potential dietary-based intervention via AR-TMPRSS2 transcriptional inhibition of critical host factors in the treatment or prevention of COVID-19 at virus entry level.

In terms of immune pathogenesis, the severity and clinical outcomes of COVID-19 patients are due to not only the viral load, but also to the host’s response that is triggered by viral entry and replication [17]. COVID-19 infection is characterized as a pro-inflammatory status by high levels of inflammatory factors produced by hyperactive immune cells. The inflammatory factors include cytokines such as IL-6, TNF-α, and G-CSF and chemokines such as CCL2, CCL1/2, and CXCL-2. These, together with reactive oxygen species, have been recognized to induce acute respiratory disease syndrome (ARDS), leading to lung fibrosis and possibly death [15, 16]. Therefore, systematically alleviating this hyper-activated inflammatory state is crucial to improve the prognosis and outcome of COVID-19 [17]. Several approaches such as IL-6 inhibitors and immuno-checkpoint inhibitors have been proposed to counteract the cytokine storm present in COVID-19 patients. However, the benefits, dose, and duration of these approaches remains to be validated [42]. A massive infiltration of mononuclear cells has also been detected in infected lungs, with parallel low levels of hyperactive T cells in circulation [16]. The immense migration of innate immune cells to the infected tissue, in order to control the viral replication, could contribute to the tissue damage and lead to multiple organ failures [43]. The immune system develops multiple mechanisms in order to control the excessive immune activation, including induction of an inhibitory receptor, production of anti-inflammatory factors, and expansion of regulatory cells, et al [44]. Meanwhile, MDSCs are a group of regulatory cells known to have the remarkable capability to regulate inflammatory responses and suppress T cell responses [45]. MDSCs have also been described to be highly increased in COVID-19 patients [31]. An emerging study further revealed that PMN-MDSCs expanded during the early stages of COVID-19 and were correlated with IL-1β, IL-6, IL-8, and TNF-α plasma levels, particularly in patients who required intensive care treatments, suggesting new therapeutic options geared towards MDSCs [32].

Emerging discussions and studies are referring to β-glucan as an efficient, low-cost, and safe way to overcome the hyper-inflammatory status while balancing effective immune responses [46, 47]. In fact, β-glucan has been widely shown to exert antiviral properties and decrease the severity of both upper and lower respiratory tract viral infections in both animal and human studies [48, 49]. Following exposure to β-glucan, innate immune cells undergo reprograming that results in immune enhancement by the activation of NK cells and CD4^+^ Th1 cells and suppression of the inflammatory response via downregulation of pro-inflammatory factors such as IL-6, CCL2, CXCL10, et al. [23]. β-glucan was reported to enhance anti-cancer immunity by suppressing MDSCs [24, 25]. Our clinical phase I trial in prostate cancer patients also indicated that oral dietary WBM reduced the counts of MDSCs [6]. In the line of evidence, we hypothesized that dietary WBM may display integrative immunoregulatory effects by suppressing pro-inflammatory cytokines, as well as MDSCs, through β-glucans.

To test our hypothesis, we demonstrated (Fig. 4) that WBM or LT predominantly suppressed a panel of cytokines when compared to Control. The cytokine signatures associated with treatments of WBM and LT greatly overlapped (15 out of 26 for WBM, 15 out of 15 for LT). More importantly, the unbiased GSEA analysis suggested that the shared cytokines included molecules that were associated with the Lung Fibrosis Pathway and the COVID-19 Adverse Outcome Pathway. These cytokines, such as IL-6, IL-7, CCL-2/MCP-1, CCL-3/CCL-4 MIP-1α/β, TNF-α, and G-CSF, have been implicated in COVID-19 pathogenesis [29, 30]. As expected, when we characterized MDSCs in both blood and spleen of WBM or LT-treated mice (Fig. 5), we observed the regulatory trend that both WBM and LT decreased the total counts of M-MDSCs and PMN-MDSCs, in peripheral blood and spleen. Such evidence supports the fact that β-glucan is the major component in WBM, acting as an immunomodulator. Besides, we also observed a subset of 11 factors which was selectively regulated by WBM. Among these, we pointed out a defined cytokine trait (CCL-12/MCP-5, CCL-19/MIP-3β, CXCL-2/MIP-2, et al) of severe COVID-19 outcome [30]. In considering that WBM is a mixture of multiple components, we cannot rule out the possibility that additional chemicals beside β-glucan in WBM may potentially exert immunoregulatory activities.

In conclusion, COVID-19 is a respiratory and systemic disorder accompanied by SARS-CoV-2 entry into host cells and rapid replication. Virus infection triggers immune dysregulation and a cytokine storm, ultimately leading to a range of symptoms along the clinical spectrum that include asymptomatic or mild respiratory symptoms, severe lung injury, multi-organ failure, and death [14 ~ 17]. Although specific target molecules and agents can act on each step of pathogenesis, in considering the complexity of the immune system and its systemic response, such interventions may be efficacious but come along with adverse reactions [50]. WBM is composed of a variety of chemical ingredients. Our preclinical studies have revealed that WBM is a unique food that can suppress TMPRSS2 expression through its anti-androgenic activity mediated through conjugated linolic acid [5] and promote an anti-inflammatory response, possibly by β-glucan. We hereby propose WBM consumption as a potential efficient, low-cost, and safe dietary approach to mitigate COVID-19. Our completed phase 1 trial using WBM determined that a dose level of up to 14 g WBM powder (equal to 140g fresh WBM)/d resulted in minimal side effects, mostly limited to grade 1 abdominal bloating. Therefore, WBM intervention is considered safe with a demonstrated mean compliance of 98.6% [6]. However, clinical studies and trials will be needed to prove its efficacy against COVID-19.

## Materials And Methods

### Mushroom and Chemical Regents

White button mushroom was processed as previously described. Briefly, 6 g of freeze-dried WBM powder generated from 60 g fresh mushrooms was boiled in 1 L hot water for 3 hours. The broth was centrifuged at 3000 g for 30 minutes, twice, to collect the fraction of supernatant. The liquid fraction was rotor-evaporated to dryness and then re-dissolved in 1 mL of hot water to produce a 6X mushroom. Therefore, the concentration of 6X WBM originated from 6 g dried WBM powder/mL (6 mg/μL) [5]. Enzalutamide, an androgen receptor antagonist, was purchased from Selleckchem (MDV3100, Selleckchem, Houston, TX). Lentinan, a shiitake mushroom-derived immunomodulatory β-(1, 3)-glucan, was purchased from Carbosynth (FL33321, Compton, Berkshire, UK).

### Animal Experiment

Eight-weeks-old male C57BL/6 mice (The Jackson Laboratory, Sacramento, CA) were used for the evaluation of WBM-mediated AR-TMPRSS2 suppression and immunomodulation. Experiments performed in this study comply with current laws in the United States of America. All applicable institutional guidelines for the care and use of animals were followed. Animal research procedures were approved by the Institutional Animal Care and Use Committee (IACUC) at City of Hope (IACUC-15091). Facilities are credited by AAALAC (Association for Assessment and Accreditation of Laboratory Animal Care) and operated according to NIH guidelines.

To study WBM-mediated AR-TMPRSS2 suppression, in each experiment, 12 mice were subcutaneously grafted with DHT pellets (12.5 mg/60 days, Innovative Research of America, Sarasota, FL), while 3 mice were grafted with placebo pellets (Innovative Research of America, Sarasota, FL). The mice with placebo pellets were gavaged daily with 100 μL PBS with 1% carboxymethyl cellulose for 2 weeks. The 12 mice with DHT pellets were randomly divided into 3 groups and treated daily for 2 weeks as follows: 3 mice in Control (Ctrl) were gavaged with 100 μL PBS with 1% carboxymethyl cellulose, 3 mice in enzalutamide (Enza) group were gavaged with 300 μg/mice enzalutamide (at a dose of 10 mg/kg), and 6 mice in WBM group were gavaged with at a dose of 200 mg/kg/day (average body weight of mice is 30 g, equal to 6 mg/mice/day) in 100 μL PBS with 1% carboxymethyl cellulose.

To study WBM-mediated immunomodulation, 15 C57BL/6 mice were randomly divided into 3 groups and treated daily for 2 weeks as follows: 5 mice in Ctrl group were gavaged with 100 μL PBS with 1% carboxymethyl cellulose, 5 mice in Lentinan (LT) group were gavaged with 6 mg/mice Lentinan at a dose of 200 mg/kg (average body weight of mice is 30 g, equal to 6 mg LT/mice/day), and 5 mice in WBM group were gavaged at a dose of 200 mg/kg (equal to 6 mg WBM powder/mice/day in 100 μL PBS with 1% carboxymethyl cellulose.

Throughout the treatments, body weights were monitored every two days as an indicator of the mice’s overall health. At the end of the treatments, the mice were euthanized. Putative COVID-19 impacted organs (lungs, kidneys, and small intestine) and androgen responsive organs (prostate) were flash-frozen in liquid nitrogen and/or fixed with 4% paraformaldehyde. The expression levels of AR, TMPRSS2, and ACE2 were assessed by qRT-PCR and immunohistochemistry (IHC). Whole blood samples were collected and isolated into serum and cell pellets. Serum samples were stored at − 80°C for the cytokine array assay, while cell pellets were pre-flxed and stored at − 80°C for the flow cytometry assay. Spleens were collected to isolate splenocytes, which were then pre-flxed and stored at − 80°C for the flow cytometry assay.

### Quantitative real-time PCR

Total RNA was extracted using the RNeasy Mini kit (QIAGEN, Germantown, Maryland) and was then used to synthesize cDNA with SuperScript IV VILO Master Mix (Thermo-Fisher, Grand Island, NY). qPCR was performed using CFX Connect Real-Time System (Bio-Rad, Hercules, California) with PerfeCTa SYBR Green FastMix (Quantabio, Beverly, Massachusetts). The primers used in the qRT-PCR were obtained from Primer Bank (https://pga.mgh.harvard.edu/primerbank). The target gene mRNA level was normalized to GAPDH expression.

### Immunohistochemistry and Histological analysis

Hematoxylin and eosin (H&E) staining and immunohistochemistry (IHC) on formalin-fixed tissues were performed by the Pathology Core at City of Hope. Antibodies used in IHC included: AR (SP107, Sigma-Aldrich), ACE2 (ab108252, Abcam), and TMPRSS2 (ab92323, Abcam). Slides were first reviewed at 10X magnification to identify areas of positive staining, followed by confirmation and quantification at 20X magnification. IHC staining was scored by QuPath software (version 0.2). AR, ACE2, and TMPRSS2 were scored by the percentage of positive cells. Representative images were acquired using an Olympus BX46 microscope with a DP27 camera at magnifications of 20X and 40X, with a scale bar of 200 μm, and 100 μm, respectively.

### Distribution and Expression Analysis in Human Tissues

For tissue distribution of mRNA and protein expressions, data on the target genes were obtained from “The Tissue Atlas” category of “The Human Protein Atlas/HPA” (http://www.proteinatlas.org/) [51]. The mRNA expression from the “The Genotype-Tissue Expression/GTEx Dataset” was chosen for demonstration with reference to the normalized consensus dataset Immunohistochemistry staining was performed on normal human tissue samples. AR expression was detected with a rabbit antibody (1:250, HPA004733, Sigma-Aldrich) and validated with another rabbit antibody (1:500, GTX62599, GeneTex). ACE2 expression was primarily detected with a rabbit antibody (1:250, HPA000288, Sigma-Aldrich) and confirmed with a mouse antibody (1:5000, CAB026174, R&D Systems); TMPRSS2 expression was detected with a rabbit antibody (1:300, HPA035787, Sigma-Aldrich). The information on expression intensity and specific cell types that express respective genes were extracted from the staining reports for each staining type in the database.

### Cytokine Profiling Assay and Data Analysis

Proteome Profiler Mouse XL Cytokine Array Kit (ARY028, Bio-techne, Minneapolis, MN), a membrane-based antibody array, was applied to semi-quantify 111 mouse cytokines, chemokine growth factors, and other soluble proteins in serum. The assay was performed following the manual’s protocol. The image of dot spots was analyzed by Quick Spots software (HLImage++, Western Vision Software, Salt Lake City, UT), which measured the mean spot pixel density. The significant changes (treatments versus control) were defined as up-regulated (log2 fold change ≥ 0.5, p < 0.05) versus down-regulated (log2 fold change ≤ 0.5, p < 0.05). The overlapping cytokines between the two treatments were displayed by Venn diagram using Venny 2.1. Gene Set Enrichment Analysis (GSEA) was used to identify pathways underlying cytokine synergism. The Top 5 pathways were presented with False Discovery Rate/FDR q values (< 0.05), p values (< 0.05) and k/K Value [Genes in Overlap (k) to Genes in Gene Set (K)].

### Flow-Cytometry Assay

BD Accuri C6 Plus flow cytometer (BD, San Jose, CA) was used to identify MDSCs in spleen and blood. BD Accuri C6 system software was used to analyze the data. Mouse MDSCs were identified by staining with the following panel of antibodies: FITC conjugated anti-mouse CD45 (30-F11, eBioscience, San Diego, CA), PerCP-Cy5.5 conjugated anti-mouse CD11b (M1/70, eBioscience, San Diego, CA), and PE conjugated anti-mouse Gr-1 Ly6G/Ly-6C (RB6–8C5, eBioscience, San Diego, CA). Monocytic MDSCs (M-MDSCs/CD45^+^/CD11b^+^/Gr-1^low/mid^) and granulocytic MDSCs (PMN-MDSCs/CD45^+^/CD11b^+^/Gr-1^high^) were gated to show the two distinct populations of MDSCs.

### Statistical Methods and Data Analysis

Results are shown as means ± standard deviation.. All statistical analyses were performed using GraphPad Prism software (version 8.0). The significance of the differences between the mean values was determined by multiple Student’s t-tests. p values < 0.05 (* p < 0.05, **p < 0.01, ***p < 0.001) were considered statistically significant and all tests were two-tailed.

## Supplementary Material

Supplement

## Figures and Tables

**Table 1.** Top 5 pathways suggested by Gene Set Enrichment Analysis (GSEA). 28 WBM-regulated cytokines (including 17 common regulated factors between WBM and LT) were analyzed by GSEA and WIKI Pathways Gene Sets were selected. Top 5 pathways were presented with False Discovery Rate/FDR q values (< 0.05), p values (< 0.05) and k/K Value [Genes in Overlap (k) to Genes in Gene Set (K)].

Gene Set Name (WIKI Pathways)		LUNG FIBROSIS	COVID19 ADVERSE OUTCOME PATHWAY	IL18 SIGNALING PATHWAY	CYTOKINES&INFLAMMATORY RESPONSE	TOLLLIKE RECEPTOR SIGNALING PATHWAY

p-Value		1.21 e-24	3.27 e-16	2.68 e-15	1.49 e-14	1.92 e-10
FDR q-Value		7.11 e-22	9.59 e-14	5.24 e-13	2.19 e-10	1.87 e-8

k/K Value		11/63	6/15	10/279	6/26	5/104

Cytokine symbol	IL-6					

	TNF-α					

	CCL-2/MCP-1					

	CCL-3/ MIP-1α					

	G-CSF					

	CXCL-2/MIP-2					

	MMP9					

	CCL-4/MIP-1β					

	Pentraxin 3					

	HGF					

	FGF acidic					

	IL-7					

	CCL19/MIP-3β					

	CCL20/MIP-3α					

	IL-11					

	CD40/TNFRSF5					
