# Supplementary figures and images for: White Button Mushroom (Agaricus bisporus) Interrupts Tissue AR-TMPRSS2 Expression and Attenuates Pro-inflammatory Cytokines in C57BL/6 Mice: Implication for COVID-19 Dietary Intervention
Source: Res Sq. 2021 Mar 25:rs.3.rs-244245. Preprint. [Version 1] doi: 10.21203/rs.3.rs-244245/v1 (PMC8010737; doi:10.21203/rs.3.rs-244245/v1)

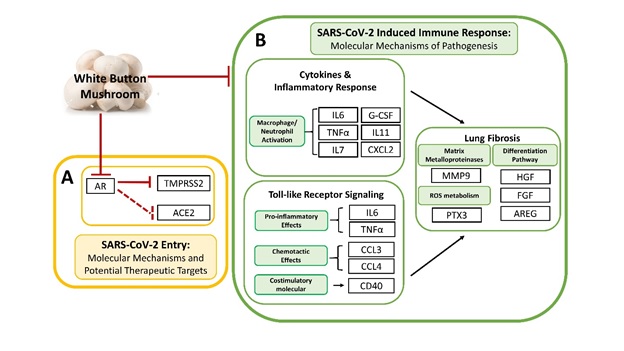

Supplement: Supplement [file 9e1c34a0fe77574f2ba2e94b.jpg]
